# Tuberculosis in the aftermath of the 2010 earthquake in Haiti

**DOI:** 10.2471/BLT.14.145649

**Published:** 2015-05-01

**Authors:** Serena P Koenig, Vanessa Rouzier, Stalz Charles Vilbrun, Willy Morose, Sean E Collins, Patrice Joseph, Diessy Decome, Oksana Ocheretina, Stanislas Galbaud, Lauren Hashiguchi, Julma Pierrot, Jean William Pape

**Affiliations:** a Haitian Study Group for Kaposi’s Sarcoma and Opportunistic Infections (GHESKIO), 33 Boulevard Harry Truman, BP 15727, Port-au-Prince, Haiti.; b Haitian National Tuberculosis Program (Programme National de Lutte contre la Tuberculose, PNLT), Port-au-Prince, Haiti.; c Department of Medicine, Stanford University, Palo Alto, United States of America (USA).

## Abstract

**Problem:**

In 2010, Haiti sustained a devastating earthquake that crippled the health-care infrastructure in the capital city, Port-au-Prince, and left 1.5 million people homeless. Subsequently, there was an increase in reported tuberculosis in the affected population.

**Approach:**

We conducted active tuberculosis case finding in a camp for internally displaced persons and a nearby slum. Community health workers screened for tuberculosis at the household level. People with persistent cough were referred to a physician. The National Tuberculosis Program continued its national tuberculosis reporting system.

**Local setting:**

Even before the earthquake, Haiti had the highest tuberculosis incidence in the Americas. About half of the tuberculosis cases occur in the Port-au-Prince region.

**Relevant changes:**

The number of reported tuberculosis cases in Haiti has increased after the earthquake, but data are too limited to determine if this is due to an increase in tuberculosis burden or to improved case detection. Compared to previous national estimates (230 per 100 000 population), undiagnosed tuberculosis was threefold higher in a camp for internally displaced persons (693 per 100 000) and fivefold higher in an urban slum (1165 per 100 000). With funding from the World Health Organization (WHO), active case finding is now being done systematically in slums and camps.

**Lessons learnt:**

Household-level screening for prolonged cough was effective in identifying patients with active tuberculosis in this study. Without accurate data, early detection of rising tuberculosis rates is challenging; data collection should be incorporated into pragmatic disease response programmes.

## Résumé

**Problème:**

En 2010, Haïti a été frappé par un séisme dévastateur qui a ébranlé les infrastructures sanitaires de la capitale, Port-au-Prince, et laissé 1,5 millions de personnes sans abri. On a par la suite observé une augmentation des cas de tuberculose recensés dans la population concernée.

**Approche:**

Nous avons mené une recherche active des cas de tuberculose dans un camp de personnes déplacées ainsi que dans un bidonville voisin. Les agents de santé communautaires ont recherché les cas de tuberculose dans les foyers. Les personnes qui présentaient une toux persistante ont été orientées vers un médecin. Le système national de signalement des cas de tuberculose a été maintenu, dans le cadre du programme national de lutte contre la tuberculose.

**Environnement local:**

Déjà avant le séisme, Haïti présentait la plus forte incidence de tuberculose d’Amérique. Près de la moitié des cas de tuberculose sont recensés dans la région de Port-au-Prince.

**Changements significatifs:**

Le nombre de cas de tuberculose signalés en Haïti a augmenté depuis le séisme, mais les données limitées ne permettent pas de déterminer si cela est dû à une augmentation de la charge de la maladie ou à une amélioration du dépistage. Comparés aux précédentes estimations nationales (230 cas pour 100 000 personnes), les cas de tuberculose non diagnostiqués étaient trois fois plus importants dans le camp de personnes déplacées (693 cas pour 100 000 personnes) et cinq fois plus dans le bidonville (1165 cas pour 100 000 personnes). Grâce à un financement de l'Organisation mondiale de la Santé, un dépistage actif est désormais effectué systématiquement dans les camps et les bidonvilles.

**Leçons tirées:**

Dans cette étude, la recherche d'une toux prolongée dans les foyers a permis d'identifier les patients atteints de tuberculose active. Sans données précises, il est difficile de détecter précocement une augmentation du taux de tuberculose ; un travail de collecte de données devrait être intégré aux programmes pratiques de réponse aux maladies.

## Resumen

**Problema:**

En 2010, Haití sufrió un devastador terremoto que movilizó a toda la infraestructura sanitaria para socorrer a la capital, Puerto Príncipe, y dejó a 1,5 millones de personas sin hogar. Además, se produjo un aumento de los casos de tuberculosis notificados entre la población afectada.

**Enfoque:**

Se llevaron a cabo detecciones de casos activos de tuberculosis en un campamento para personas desplazadas de la ciudad y en un barrio pobre cercano. Los agentes de salud comunitarios investigaron los casos de tuberculosis a nivel doméstico. Las personas con tos persistente fueron derivadas a un médico. El programa nacional contra la tuberculosis continuó su sistema nacional de informes sobre la tuberculosis.

**Contexto local:**

Incluso antes del terremoto, Haití tenía el mayor número de casos de tuberculosis de todo el continente americano. Alrededor de la mitad de los casos de tuberculosis se encontraba en la región de Puerto Príncipe.

**Cambios pertinentes:**

El número de casos registrados de tuberculosis en Haití aumentó tras el terremoto, aunque no existen los suficientes datos para determinar si este suceso se debe a un aumento de la carga de tuberculosis o a un mejor sistema de detección de casos. En comparación con los cálculos nacionales (230 de cada 100.000 personas), la tuberculosis sin diagnosticar era el triple en un campamento para personas desplazadas de la ciudad (693 de cada 100.000 personas) y cinco veces mayor en el barrio pobre urbano (1.165 de cada 100.000 personas). Con fondos de la Organización Mundial de la Salud, actualmente se realiza una búsqueda de casos activos de forma sistemática en barrios pobres y campamentos.

**Lección aprendida:**

La búsqueda a nivel doméstico de tos persistente resultó eficaz a la hora de identificar a pacientes con tuberculosis activa en este estudio. Sin datos precisos, la detección temprana de mayores tasas de tuberculosis supone todo un reto. Los datos recogidos se incorporarán a programas de respuesta pragmática contra enfermedades.

## ملخص

**المشكلة:**

عانت دولة هايتي في عام 2010 من زلزال مدمر أصاب المرافق الأساسية للرعاية الصحية في العاصمة "بورت أو برانس" بالشلل، مخلفًا 1.5 مليون نسمة بلا مأوى. وأدى ذلك إلى ارتفاع في معدل تسجيل حالات الإصابة بالسل في المجموعة السكانية المنكوبة.

**الأسلوب:**

أجرينا دراسة للكشف عن حالات الإصابة الفعلية بالسل في مخيم للمشردين داخل البلاد وأحد الأحياء الفقيرة المجاورة. كما أجرى العاملون بمجال الخدمات الصحية المجتمعية فحصًا للكشف عن حالات الإصابة بالسل على المستوى الأسري. وتمت إحالة المصابين بسعال مستمر إلى أحد الأطباء. واستمر البرنامج القومي لمواجهة السل في اتباعه النظام القومي لتسجيل حالات الإصابة بالمرض.

**المواقع المحلية:**

كانت دولة هايتي تسجل أعلى معدل للإصابة بمرض السل في الأمريكيتين حتى قبل وقوع الزلزال. وقد وقعت نصف حالات الإصابة بمرض السل في منطقة "بورت أو برانس".

**التغيرات ذات الصلة:**

ارتفع عدد حالات تسجيل الإصابة بمرض السل في دولة هايتي بعد وقوع الزلزال، ولكن البيانات المتاحة عن ذلك محدودة جدًا بما لا يسمح بتحديد ما إذا كان الأمر يرجع إلى زيادة عبء السل أم إلى تحسن أساليب اكتشاف حالات الإصابة بالمرض. ومقارنةً بالتقديرات التي أجريت على المستوى القومي (230 شخص بين 100,000 شخص)، بلغت حالات الإصابة بالسل التي لم يتم تشخيصها نسبة تزيد بثلاثة أضعاف في مخيم الأشخاص المشردين على المستوى المحلي (693 شخصًا بين 100,000 شخص) وتزيد بخمس أضعاف في أحد الأحياء الحضرية الفقيرة (1165 شخص بين 100,000 شخص). وبفضل تمويل منظمة الصحة العالمية، تُجرى الآن دراسة للكشف عن حالات الإصابة الفعلية بالسل على نحو منهجي في الأحياء الفقيرة والمخيمات.

**الدروس المستفادة:**

كان للفحص على المستوى الأسرى لاكتشاف حالات الإصابة بالسعال المستمر لفترة طويلة أثرٌ فعال في تحديد المرضى المصابين فعليًا بالسل في هذه الدراسة. وسيصبح الكشف عن الارتفاع في معدلات الإصابة بالسل تحديًا صعبًا في غياب البيانات الدقيقة؛ ومن ثمّ يجب تضمين أسلوب جمع البيانات في البرامج الواقعية لمواجهة المرض.

## 摘要

**问题:**

2010 年，海地经历了一次破坏性大地震，导致其首都太子港的医疗保健基础设施陷入瘫痪，150 万民众无家可归。随后，据报告受地震影响人群中的结核病患者数量增加。

**方法:**

我们在收容境内流离失所人员的帐篷和周边的贫民区中查找活动性结核病病例。社区卫生工作人员挨家挨户筛查结核病患者。嘱咐长期咳嗽人员就医。国家结核病规划延续了其国家结核病报告系统。

**当地状况:**

在地震发生之前，海地就是美洲结核病发病率最高的国家。大约有一半的结核病病例发生在太子港地区。

**相关变化:**

地震之后，海地报告的结核病病例数量有所增加，但是数据有限，无法确定是由于结核病总量增加还是病例检测技术改进所引起的。与之前的国家估计数据（每 100 000 人中 230 人）相比，收容境内流离失所人员的帐篷内未确诊的结核病患者高出 3 倍（每 100 000 人中 693 人）,城市贫民区中未确诊的结核病患者高出 5 倍（每 100 000 人中 1165 人）。在世界卫生组织的资助下，目前正系统地在贫民区以及帐篷中查找活动性病例。

**经验教训:**

在本次研究中，挨家挨户对长期咳嗽的人员进行筛查是确认活动性结核病患者的有效方法。没有准确的数据，结核病率上升的早期检测困难重重；应将数据收集纳入务实的疾病应对规划。

## Резюме

**Проблема:**

В 2010 году на Гаити произошло разрушительное землетрясение, которое нанесло значительный ущерб инфраструктуре здравоохранения в столице страны Порт-o-Пренс и оставило без крова 1,5 миллиона людей. В результате возросло количество зарегистрированных случаев туберкулеза среди населения пострадавших от землетрясения районов.

**Подход:**

Была проведена активная диагностика случаев заболевания туберкулезом в лагере вынужденных переселенцев и ближайшем районе трущоб. Местные работники здравоохранения выполняли скрининговое обследование на туберкулез в каждой семье. Люди, страдающие от постоянного кашля, были направлены к врачу. Национальная программа по борьбе с туберкулезом продолжала вносить результаты работы в национальную систему отчетности.

**Местные условия:**

Даже до землетрясения уровень заболеваемости туберкулезом на Гаити был одним из самых высоких в странах Северной и Южной Америки. Около половины случаев заболевания туберкулезом регистрировалось в районе Порт-o-Пренс.

**Осуществленные перемены:**

Количество зарегистрированных случаев заболевания туберкулезом на Гаити возросло после землетрясения. По причине недостаточности данных сложно определить, является ли рост заболеваемости результатом и без того высокого уровня заболеваемости туберкулезом в стране или результатом более совершенного процесса выявления случаев заболевания. В сравнении с предыдущими оценками на национальном уровне (230 случаев на 100 000 человек населения) было выявлено в три раза больше недиагностированных случаев заболевания туберкулезом в лагере вынужденных переселенцев (693 случая на 100 000 человек) и в пять раз больше таких случаев в районах городских трущоб (1165 на 100 000 человек). Благодаря финансированию Всемирной организации здравоохранения в лагерях и городских трущобах теперь систематически проводится активная диагностика случаев заболевания.

**Выводы:**

Скрининговое обследование по жалобам на постоянный кашель в семьях, проводившееся в рамках данного исследования, оказалось эффективным для выявления пациентов с активной формой туберкулеза. Раннее выявление растущего числа заболеваний туберкулезом затруднительно по причине недостаточности данных. Сбор данных необходимо включить в практические программы противодействия распространению заболевания.

## Introduction

On January 12, 2010, Haiti sustained a devastating earthquake. Total damages are estimated at 7.8 billion United States dollars, more than 120% of the country’s 2009 gross domestic product.^1^ Over 1.5 million people lost their homes and about 279 000 remained internally displaced in tent camps nearly four years later.^2^ Even before the earthquake, Haiti had the poorest economic and health indices in the region of the Americas.^3^ Haiti also had the highest tuberculosis incidence in the Americas (230 per 100 000 population in 2010) – nearly 10-fold higher than the regional incidence of 30 per 100 000 – and higher than the overall incidence of the world’s 22 high-burden countries (166 per 100 000).^4^ About half of the tuberculosis cases in Haiti occur in the West Department that includes Port-au-Prince and that was most heavily affected by the earthquake. Government buildings and health-care centres were destroyed, including the building that housed the National Tuberculosis Program, the two largest tuberculosis sanatoria and many clinics. Though most clinics resumed services within months, thousands of patients were initially dispersed in camps without tuberculosis medication.

Typically, tuberculosis rates remain stable in the immediate aftermath of natural disasters.^5^^–^^7^ In Haiti, however, the internally displaced persons camps were crowded, the sanitation was poor, the children were chronically malnourished and the duration of residence was often prolonged.^8^ The physical, social and economic damage to an already limited health-care infrastructure worsened the risk for acquiring active tuberculosis, hampered surveillance and challenged the public health response. The National Tuberculosis Program worked to open tuberculosis clinics as quickly as possible and the Haitian Group for the Study of Kaposi’s Sarcoma and Opportunistic Infections (GHESKIO) conducted active case finding in a camp and slum adjacent to the clinic using cough as an indicator for tuberculosis. Studies done over the prior decade have shown that about one-third of patients presenting for human immunodeficiency virus (HIV) testing with chronic cough had active tuberculosis.^9^^,^^10^

**Fig. 1:**
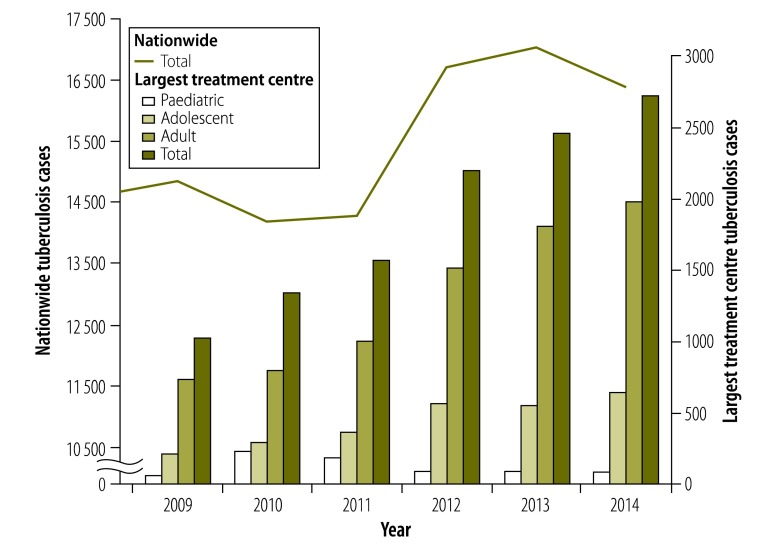
Number of persons diagnosed with tuberculosis, Haiti, 2009–2014

Note: Data for 2014 are preliminary pending final reporting from the National Tuberculosis Program. Paediatric represents the age range: < 10 years; adolescent represents the age range:10–23 years.

At the largest treatment centre in Haiti, the annual number of tuberculosis cases has more than doubled since the earthquake. The increase was first noted in paediatric patients. In 2010, 242 children younger than 10 years were diagnosed with tuberculosis, compared to 72 in 2009, a 336% increase. Fifty-two percent of cases were children younger than two years and 33% were two to five year-olds. Clinicians were concerned that this rise in paediatric cases indicated an increase in ongoing transmission from adults to children, as children are likely to develop active disease soon after exposure. Higher numbers of paediatric cases were followed by a progressive rise in adolescent and adult tuberculosis cases, from 1026 in 2009, to 2719 in 2014 (Fig. 1). Meanwhile, the total number of patient visits increased by only 5%, from 246 276 in 2009 to 258 089 in 2013.

### Active case finding

After the earthquake, a tent camp was constructed for the neighbourhood residents who lost their homes. This camp housed 5913 people when we started collecting data, and the number of people declined over time as residents were relocated to permanent dwellings.^11^ Community health workers screened camp residents in their tents and referred those reporting a cough of more than two weeks’ duration for physician evaluation with smear microscopy and a chest radiograph. From July 2010 to June 2011, 282 patients were evaluated and 34 diagnosed with pulmonary tuberculosis; 22 (65%) were sputum smear-positive (Table 1). Seven patients with tuberculosis were younger than eight years and five of these children had a parent sharing the same tent subsequently diagnosed with active tuberculosis. The estimated tuberculosis incidence in the camp was 693 cases per 100 000 person-years, about three times the 2010 World Health Organization estimate for Haiti.^3^

**Table 1:** Outcomes of active case finding for tuberculosis in a camp for internally displaced persons and a slum in Port-au-Prince, Haiti, 2010–2013

Location	Time period	Residents identified with cough ≥ 2 weeks, No.	Patients receiving sputum microscopy, No. (%)		Cases of pulmonary tuberculosis,^a^ No.	Sputum smear-positive cases,^a^ No. (%)	Incidence of tuberculosis/ 100 000 person-years
Internally displaced persons camp	1 July 2010 to 30 June 2011	282		176 (62%)	34	22 (65)	693
Cité de Dieu Slum	17 August 2011 to 16 August 2013	1420		unknown^b^	233	183 (79)	1165

^a ^Tuberculosis cases were either smear-positive, or diagnosed by a combination of symptoms and chest radiograph findings.

^b ^It is unknown how many of the 1420 coughing patients had smear microscopy. Of the 233 patients with active TB, 212 had a sputum smear.

In August 2011, using the same strategy, we expanded active tuberculosis case finding into a section of the Cité de Dieu slum, which has about 10 000 residents. From August 2011 to August 2013, 1420 patients were evaluated and 233 diagnosed with pulmonary tuberculosis; 183 (79%) were sputum smear-positive (Table 1). The estimated tuberculosis incidence was 1165 cases per 100 000 person-years, more than five times WHO’s 2010 estimate for Haiti.^3^^,^^4^

Active case finding efforts are now being expanded to over 220 000 people in slums and camps in Port-au-Prince, with a grant from WHO, through a collaboration between the National Tuberculosis Program, the largest treatment centre and the Haitian National Laboratory. Community health workers are conducting systematic door-to-door surveillance to identify people with persistent coughs and refer them to a tuberculosis clinic for diagnosis and treatment. The targeted population meet WHO’s criteria for systematic tuberculosis screening: poor socioeconomic conditions and a high prevalence of tuberculosis.^12^ Data from this surveillance will be evaluated independently by WHO and will provide further evidence of the burden of tuberculosis in Haiti.

### Changes in reported tuberculosis

Despite the loss of their offices, the National Tuberculosis Program officers continued their tuberculosis reporting system after the earthquake. Each tuberculosis clinic in Haiti provides quarterly data that the National Tuberculosis Program tallies and submits to WHO. In 2010, the National Tuberculosis Program reported a 4% (from 14 861 to 14 222) decrease in tuberculosis cases nationwide and 9.5% (from 6489 to 5871) decrease in the West Department. This apparent decline is probably due to incomplete reporting after the earthquake. Compared with 2009 (*n* = 6489), the number of cases in the West Department increased by 7% (*n* = 6944) in 2011 and 17% (*n* = 7596) in 2012. The number of cases nationwide increased by 19% from 2011 to 2013, from 14 315 to 17 040 (Fig. 1); preliminary data indicate a slight decrease to 16 400 cases in 2014.^4^

Though HIV infection is an important risk factor for the development of tuberculosis, the increase in tuberculosis cases is not explained by changes related to HIV. The proportion of tuberculosis patients who are HIV-infected has remained stable at 20% and the estimated prevalence of HIV has been stable at 2.2%.^3^^,^^4^^,^^13^ Haiti’s response to HIV is likely to be responsible for the lack of increase in HIV-associated tuberculosis after the earthquake. Although many HIV clinics were damaged in the earthquake and testing declined in 2010, the annual number of HIV tests rose above pre-earthquake levels by 2011 and 86% of tuberculosis patients were tested for HIV in 2013, compared to the regional average of 69%.^4^^,^^14^ Within months, 90% of the pre-earthquake patients had resumed antiretroviral therapy, reducing their risk of contracting tuberculosis.^15^

### Need for improved data

With only one source of tuberculosis data for Haiti, we cannot be certain whether there is a change in prevalence, case detection or a combination of the two. There were no structural changes during this time adequate to explain an increased case detection rate. Efforts to expand active case finding had been localized and intermittent, there were no major changes in the network of tuberculosis laboratories and the proportion of new pulmonary tuberculosis cases that are bacteriologically-confirmed has not changed substantially (66% in 2010, 64% in 2011, 65% in 2012 and 68% in 2013).^4^

Better data on the epidemiology of tuberculosis in Haiti are needed to understand the true burden of disease. Tuberculosis prevalence has never been assessed from a population-based survey in Haiti and there are no recent tuberculosis mortality data from a national vital registration system. WHO is therefore left with incomplete data on which to base its tuberculosis estimates for Haiti and as a result, confidence intervals are wide; estimated tuberculosis prevalence in Haiti is 129 to 421 per 100 000, compared to 30 to 48 per 100 000 for the Americas.^3^^,^^4^ This makes short-term changes in tuberculosis rates difficult to detect. The National Tuberculosis Program is planning a thorough review of tuberculosis surveillance data. The inclusion of tuberculosis mortality data would also be useful. Periodic surveys can measure trends and guide expansion of public health interventions, including active case finding.

## Discussion

Available data point to a rise in the number of reported tuberculosis cases in Haiti after the earthquake, but data are too limited to determine if this is due to an increase in tuberculosis burden or to improved case detection. Although it is clear that active case finding efforts have identified additional patients with tuberculosis, the scale of these activities thus far does not account for this increase in cases. These findings demonstrate that tuberculosis should be monitored in post-disaster settings when the affected population lives in conditions conducive to tuberculosis transmission (Box 1). To improve tuberculosis control, better data are needed to detect changes, track trends and efficiently direct a response that focuses scarce resources on the populations with the highest tuberculosis burden.

Box 1 **Lessons learnt**

- In post-disaster settings with endemic tuberculosis, tuberculosis incidence can rise if poor living conditions foster transmission.
- Without accurate data, it is difficult to distinguish whether a rise in the reported number of cases is due to a higher burden of disease or to improvements in case detection.
- Household-level screening for cough is effective in crowded, urban settings to identify patients with active tuberculosis.

In implementing active case finding activities, we learned that screening for chronic cough in slums and camps is effective in diagnosing patients with active tuberculosis in a post-disaster setting.^9^^,^^10^ This approach, using trained community health workers to identify patients with chronic cough, is efficient, low-cost and feasible for scale-up. Future steps will include expansion of these activities in Haiti and training of community health workers to provide diagnostic and treatment services for other diseases, a strategy which is promoted by the Haitian Ministry of Health to improve the health of the population at large.
